# Gaps in care, support, and research among diverse patients with early onset cancers

**DOI:** 10.1007/s00520-025-10117-4

**Published:** 2025-12-02

**Authors:** Jamie M. Reedy, Nancy A. Borstelmann, Sakinah Suttiratana, Thejal Srikumar, Farah Fasihuddin, Michelle Zupa, Laura Gross, Veda N. Giri

**Affiliations:** 1 Yale Cancer Center, Yale School of Medicine, 333 Cedar Street WWW214A, New Haven, CT 06520 USA; 2 Yale Child Study Center, Yale School of Medicine, 350 George Street, New Haven, CT 06520 USA

**Keywords:** Early onset cancer, Psychosocial support gaps, Cancer disparities

## Abstract

**Purpose:**

Early onset cancers (EOC), defined as cancers diagnosed between the ages of 18 and 49, are on the rise. Younger adults may experience amplified cancer-related challenges. Furthermore, the unique needs of Black/African American (B/AA) and Hispanic/Latine (H/L) adults with EOC have been understudied. Here we report on results from the PROSPER study, which explored the experiences of B/AA and H/L patients with EOC.

**Methods:**

Patients diagnosed with early onset breast, gastrointestinal (GI), genitourinary, and gynecological cancers were recruited from a single institution in the Northeastern US. Between February 2024 and April 2024, data were collected from 14 women who participated in both individual interviews (B/AA = 7; H/L = 7) and focus groups (B/AA Group, *n* = 5; H/L Group, *n* = 3) in English and/or Spanish. Participants (ages 28–49) were diagnosed with breast (*n* = 7), GI (*n* = 5), ovarian (*n* = 1), or breast and GI (*n* = 1) cancer. Interviews were recorded, transcribed, and qualitatively analyzed using modified rapid analysis techniques.

**Results:**

Thematic analysis identified challenges across six psychosocial domains spanning the impact of cancer on the patient and family, limited access to information, communication concerns, financial strain, and balancing care with life activities. We also identified unique barriers within our cohort related to clinical care, support needs, and applicable research.

**Conclusion:**

Our results support existing literature on the challenges faced by patients with EOCs, while also providing critical insights into the unique experiences of B/AA and H/L women with EOCs. These results inform the development of EOC program strategies.

## Introduction

The rates of early onset cancers (EOC), defined as cancers diagnosed between ages 18 and 49, have been rising since 1990, with a 79.1% increase in incidence and a 27.7% increase in mortality across multiple cancer types, including breast, genitourinary (GU), gynecologic (GYN), and gastrointestinal (GI) cancers [1–3]. Black/African Americans (B/AA) and Hispanic/Latine (H/L) adults under 50 are diagnosed at higher rates and have lower 5-year overall survival than their Non-Hispanic White (NHW) peers [4–6].

Patients with EOC face unique cancer- and age-related challenges, often requiring enhanced support in areas such as parenting, adult caregiving, finances, fertility, and social isolation [7]. These challenges may be amplified for the underinsured or those with difficulties accessing services due to transportation, childcare, or language barriers [8, 9]. While a significant gap still exists in understanding the needs of patients with EOC more broadly, the unique needs of Black/AA and Hispanic/Latine adults with EOC remain understudied. Therefore, we engaged B/AA and H/L adults with EOC to identify and explore a range of support domains and barriers to care, aiming to develop a more comprehensive understanding of their needs and inform tailored intervention strategies.

## Methods

The PROSPER study is a qualitative study grounded in the Theory of Planned Behavior that was conducted with focused recruitment of B/AA and H/L adults diagnosed and treated for an EOC (Breast, GI, GU, and GYN) within the past 3 years at oncology clinics in one Northeastern US academic health system. Eligible participants were identified from a patient database query and sent recruitment materials. All participants provided informed consent in this Institutional Review Board-approved study.

Between February 2024 and April 2024, data were collected from 14 women who participated in both individual interviews (B/AA = 7; H/L = 7) and focus groups (B/AA Group, *n* = 5; H/L Group, *n* = 3) in English and/or Spanish. Participants (ages 28–49) were diagnosed with breast (*n* = 7), GI (*n* = 5), ovarian (*n* = 1), or breast and GI (*n* = 1) cancer. Trained, racial/ethnic-concordant facilitators conducted virtual interviews and focus groups exploring participants’ attitudes about their cancer care and experiences managing life with cancer. The facilitators’ guide was informed by existing literature and patient/family insights gleaned through collective clinician experience [7–12].

Interviews were recorded, transcribed, and analyzed using modified rapid analysis techniques to inform intervention development [13, 14]. Our thematic schema included an a priori EOC framework [7–12], with emerging concepts added during inductive coding. Focus groups provided extended narratives and contributed to the emergence of key themes. Consistent with qualitative sampling methodology, recruitment was closed after achieving thematic saturation with 14 participants over a 3-month period [15].

## Results

We identified challenges across six psychosocial domains including (1) cancer’s impact on personal life and coping; (2) cancer information access and availability; (3) challenges communicating about cancer to family and supporters; (4) balancing cancer care while managing life responsibilities; (5) cancer’s impact on family and supporters; and (6) financial stress and challenges navigating financial support and insurance. These challenges were consistent with the domains in the EOC framework and across existing literature [7–12]. See Table 1 for supporting details from participants. Furthermore, we identified unique challenges for our B/AA and H/L cohort across three additional domains: (1) clinical care, (2) support needs, and (3) applicability of available research. This report focuses on these three domains to build upon current knowledge of EOC support needs and barriers.

**Table 1:** Highlighted participant quotes from the psychosocial cancer domains in our literature-informed EOC framework [7–12]

Psychosocial cancer domain	Highlighted quote
Cancer’s impact on personal life and coping	**Participant 13** “You’re just trying to pull through. It was like being brought to a halt… the biggest challenge was adjusting to the physical, mental and emotional changes.”
Cancer information access and availability	**Participant 7** “[Patients] definitely [need] help with understanding your type of cancer…the different types of treatments and medications that go with it…different doctors and what role each doctor plays in your treatment, and surgeons…the different types of surgeries…just knowing that there are options and…different approaches that are available.”
Challenges communicating about cancer to family and supporters	**Participant 12** “Explaining the whole thing to literally all of my family…trying to figure out your own emotions to be able to clearly communicate with family members.”
Balancing cancer care while managing life responsibilities	**Participant 14** ““For my toddler, I didn’t have any caregiver. I didn’t have anybody to help me take care of her. The days that I had to go to the hospital, because I had different appointments at different times, it was so overwhelming. Sometimes I would go to the hospital with her, and I’m not supposed to bring her in because she’s a baby. And sometimes my husband will have to stop jobs, call out to just help me take care of the child. It was just a lot of challenge[s].”
Cancer’s impact on family and supporters	**Focus Group 1** “To me, [the hardest part] was preparing my family…the emotional and physical shock isn’t just for the patient, but also for the family…it all happens so fast.”
Financial stress and challenges navigating financial support and insurance	**Participant 5** “Even though you have insurance and a lot of it was covered, nobody was really clear about like, ‘Okay, well, you have insurance, but you also have a deductible and you have copays and those can add up.’ And they did. They added up a lot.”

### Clinical care challenges

Participants reported challenges accessing and understanding their care options due to limited English proficiency or low health literacy. Other racially/ethnically diverse participants perceived differences in their quality of care as compared to their NHW peers. Participants also expressed a perceived decline in both the quality of care and care team relationships if their individual or religious beliefs conflicted with standardized care practices. Refer to Table 2 for supporting details from participants.

**Table 2:** Highlighted participant quotes from three newly identified domains: Gaps in clinical care, support needs, and the applicability of available research

Theme	Sub-theme	Highlighted quotes
Care gaps	**Race/ethnicity**	• **Participant 13**: “I have a white mother. And to see the type of care she got versus the type of care I got, it blew my mind, to be honest. I kind of feel like I was blown off…You shouldn’t be treated a certain way because of the color of your skin or because you choose not to take a medication or whatever the case may be.” • **Focus Group 2:** “The fear of the diagnosis can be very overwhelming. You try to get all the support from the care team, and you don’t get that because, for so many reasons in terms of culture, they stigmatize you for race, the treatment plan and everything. They don’t want to give you their best.”
	**Language**	• **Focus Group 1:** “Just making it easier for people to go on and find all this information without having to ask or wait for someone to get back to you…when your first language is Spanish or any other language, it’s very hard…to get those services or even to know what to ask.”
	**Individual beliefs**	• **Participant 9:** “[My doctor] said, “You’re going to have surgery. You have any concerns?” And I said, “No, but I am one of Jehovah’s Witnesses, and I will not take blood. So I need to find a surgeon that is compliant with that.” And she asked me why. And I answered her. And she asked me why [*again*]. And I shared what the scripture said, and then she asked me again why…I felt she was being very pushy and not compassionate, especially when someone gets that kind of news.” • **Participant 13:** “Unfortunately, I am a smoker…They threatened to cancel my surgery if I didn’t stop smoking…[*they told me*] the scar tissue was going to turn black and my skin was going to die…they went to the extremes to scare me. My surgery actually did get canceled… didn’t need to be talked to the way that I was talked to…And I did everything I possibly could…They kept checking my nicotine levels, making me go for blood work…the way that I was treated because I was a smoker…I’m going through enough…I did stop at one point…just so I can have my surgery and everything would be okay…I left so many appointments bawling my eyes out.”
Support gaps	**Language**	• **Participant 3:** “I would have liked a group…the language barrier held me back…because I can understand English, I can speak some English, I can read English, but when it comes to holding a long conversation…So that was a barrier – not knowing if there were groups in Spanish.”
	**Peer matching**	• **Participant 14:** “Sometimes I feel some of the information might not be correct, because I know people are on social media for different reasons. Some people are there to make money, to have more traffic on their page, more people following them. So sometimes I feel the information they give out there can be confusing…because my experience…might be different from somebody else’s experience.” • **Participant 1:** “There is so much more offered for patients who have breast cancer. When it came for colon cancer, there was very [minimal]…” • **Focus Group 1:** “There’s very little talk about ovarian cancer. There’s very little information.”
	**Cultural concordance**	• **Focus Group 2**: “I had lots of challenges…regarding food…a lot of food from the pantry that was not cultural[ly] related to me and my kids…I couldn’t have most of the food…”
Research gaps	**Applicability of research**	• **Participant 2:** “They were extra interested in my genetics being that I was so young [*and*] Black…They don’t have any research for a 40-year-old Black female, so none of what you’re reading pertains to you…” • **Participant 5:** “There was no information for a 40-year-old Black woman with pancreatic cancer. Everything was for 70-year-old white men, and all that information was morbid.”

^a^ Participant IDs were assigned in the order in which interviews were scheduled. Some quotes have been minimally edited for clarity

### Support challenges

Participants described gaps in access to cancer-related information and resources due to language and cultural barriers. Many also found it challenging to connect with peers across similar cancer experiences, age, and racial/ethnic identities. While some individuals supplemented connections through social media, there was hesitation in trusting information shared on these platforms. See Table 2 for detailed patient experiences.

### Research gaps

Participants shared frustration when care teams indicated that available research or cancer information may not apply to them due to race/ethnicity, age, EOC type, or a combination of these factors. Consequently, participants expressed uncertainty about shared decision-making with their cancer team due to existing research gaps (Table 2).

## Discussion and implications

Our findings aligned with EOC experiences reported in the existing literature used to create our EOC framework, and encompassed challenges across six psychosocial domains spanning the impact of cancer on the patient and family, limited access to information, communication concerns, financial strain, and balancing care with life activities [7–12]. These cancer-related challenges emphasize the importance of enhanced support for patients with EOCs [7–12]. We also identified unique barriers and experiences among B/AA and H/L women with EOC, including gaps in care, such as challenges across language, race/ethnicity, and individual beliefs; support gaps ranging from a lack of language/culture-concordant supportive services to the absence of social support matching their preferred identities; and research gaps in the available data relevant to this younger B/AA and H/L cohort. These identified challenges present an opportunity to develop personalized, culturally tailored interventions for patients with EOC.

Our results contribute to an expanded understanding of the clinical, research, and support challenges faced by understudied B/AA and H/L females with EOCs and help inform the development of EOC programs. Participants strongly supported the development of a personalized digital cancer support tool with reliable, culturally tailored resources for EOCs. A customized digital EOC tool could synergize with patient-centered cancer navigation, enhancing engagement in care by targeting priority psychosocial support needs. Informed by these findings, we launched an Early Onset Cancer Program that incorporates personalized care navigation and ongoing development of a digital cancer support tool.

Recruiting males presented challenges, prompting the development of focused male recruitment strategies across subsequent study phases, including tailored outreach, peer promotion, and collaboration with trusted community influencers. Future studies should incorporate tailored recruitment strategies for individuals with diverse gender identities to foster more inclusive perspectives. Despite the small sample size, thematic saturation was achieved with 14 participants. Future studies aiming to expand the sample size will need to weigh the potential for identifying new themes important for cancer patients or survivors against the burden of participation.

In summary, the findings from the PROSPER study support the existing literature on the experiences of patients with EOCs [7–12], while also providing critical new insights into the unique challenges experienced by B/AA and H/L women. These results inform the development of EOC program strategies applicable to the needs of all patients.

## Data Availability

No datasets were generated or analysed during the current study.
